# Teprotumumab Improves Quality of Life in Thyroid Eye Disease: Meta-analysis and Matching-adjusted Indirect Comparison

**DOI:** 10.1210/jendso/bvaf063

**Published:** 2025-04-08

**Authors:** George J Kahaly, Ann Xi, Naina Barretto, Haridarshan Patel, Anahita Qashqai, Mostafa Shokoohi, Paul Spin, Robert J Holt

**Affiliations:** Department of Medicine I, Johannes Gutenberg University Medical Center, Mainz 55101, Germany; Amgen Inc., Thousand Oaks, CA 91320-1789, USA; Amgen Inc., Thousand Oaks, CA 91320-1789, USA; Amgen Inc., Thousand Oaks, CA 91320-1789, USA; Amgen Inc., Thousand Oaks, CA 91320-1789, USA; Value and Evidence, EVERSANA, Burlington, ON L7N 3H8, Canada; Value and Evidence, EVERSANA, Burlington, ON L7N 3H8, Canada; Amgen Inc., Thousand Oaks, CA 91320-1789, USA

**Keywords:** quality of life, Graves’ orbitopathy, teprotumumab, methylprednisolone, thyroid eye disease, matching-adjusted indirect comparison

## Abstract

**Context:**

Teprotumumab and/or intravenous methylprednisolone (IVMP) are first-line treatments for thyroid eye disease (TED)/Graves’ orbitopathy. Direct comparisons of these treatments on Graves’ Orbitopathy Quality of Life Questionnaire (GO-QoL) are lacking.

**Objective:**

Systematic review, meta-analysis, and matching-adjusted indirect treatment comparison (MAIC) of GO-QoL improvements.

**Methods:**

A literature search was performed using PubMed and Embase between inception to April 1, 2023, including studies of patients with moderate to severe active TED treated with teprotumumab or IVMP.

Changes in GO-QoL scores and appearance and visual function subscales were extracted. Two teprotumumab (n = 84) and placebo (n = 87) randomized clinical trials and 5 studies with IVMP (4.5 mg/12 weeks, n = 304) were identified. MAIC compared teprotumumab/placebo by adjusting for baseline GO-QoL, diplopia, proptosis, smoking, age, and sex. Significant improvements in overall GO-QoL were observed for teprotumumab vs IVMP [mean difference (MD): 13.26; 95% confidence interval (CI): 7.44, 19.09) and placebo (MD: 12.57; 95% CI: 5.94, 19.21), but not IVMP vs placebo (MD: −2.06; 95% CI: −8.24, 4.12). Improvements were noted in appearance subscale GO-QoL for teprotumumab vs IVMP (MD: 7.50; 95% CI: 0.35, 14.64) and placebo (MD: 10.80; 95% CI: 3.62, 17.98) but not for IVMP vs placebo (MD: 0.91; 95% CI: −6.36, 8.18). Visual subscale GO-QoL displayed greater improvements for teprotumumab vs IVMP (MD: 17.66; 95% CI: 7.86, 27.47) and placebo (MD: 14.54; 95% CI: 6.10, 22.99), with no significant change for IVMP vs placebo (MD: −3.65; 95% CI: −12.88, 5.57). Orbital pain, teprotumumab treatment, diplopia, proptosis, and male sex were significant independent predictors of overall GO-QoL (*P* < .05).

**Conclusion:**

This unique analysis demonstrated a more clinically meaningful improvement in the health-related quality of life, as measured by the GO-QoL, for TED patients with teprotumumab vs both the most recommended IVMP dosage and placebo.

Thyroid eye disease (TED), also known as Graves’ orbitopathy (GO), is a chronic, progressive, autoimmune disease characterized initially by inflammation of the eyes and surrounding connective tissues [1]. Progression can span mild symptoms like dry eyes, local irritation, and eyelid retraction to more severe manifestations such as proptosis, diplopia, intense pain, and optic nerve compression, which can result in vision loss [1, 2]. TED incidence has been estimated as 16 cases/100 000 population/year for women and 2.9 cases/100 000 population/year for men [3].

TED significantly impairs quality of life (QoL), particularly impacting visual function and appearance [4-8]. The disease-specific, validated Graves’ Orbitopathy Quality of Life Questionnaire (GO-QoL) measures QoL, with subscales for visual function and appearance [9]. Functional impairment disrupts daily activities such as reading, driving, computer work, and watching television, while appearance changes can lead to psychosocial challenges. This negative QoL effect is closely tied to disease activity and severity, often persisting over an extended period owing to ongoing signs and symptoms [10]. TED patients with the lowest QoL more frequently report pain behind the eyes, blurry vision, and diplopia [10].

Until recently, therapeutic options for active TED were limited. The 2021 European Group on Graves’ Orbitopathy guidelines recommend a cumulative dose of 4.5 g of intravenous methylprednisolone (IVMP) administered over 12 weeks as first-line treatment for moderate to severe active TED without diplopia and/or severe proptosis [2]. Although IVMP is frequently used for moderate to severe active TED, its effect on proptosis and diplopia improvement is variable [11, 12]. Similarly, a joint consensus issued by the American Thyroid Association and the European Thyroid Association recommends IVMP therapy for moderate to severe active TED without significant proptosis/diplopia (referred to as the standard of care) and teprotumumab for moderate to severe active TED with significant proptosis and/or diplopia [13].

In January 2020, the US Food and Drug Administration approved teprotumumab for treating TED [14], based on 2 placebo-controlled, double-blind, randomized clinical trials (RCTs) in patients with moderate to severe active TED [15, 16]. Teprotumumab is a human monoclonal antibody inhibitor of insulin-like growth factor I receptor (IGF-IR), which plays an instrumental role in the pathophysiology of TED [17-19]. The trials demonstrated significant reductions in inflammation, proptosis, and diplopia with teprotumumab over 24 weeks [15, 16]. Pooled analysis of the trials found moderate to large improvements in overall GO-QoL scores and appearance and visual function scores for teprotumumab compared to placebo [20].

No head-to-head randomized controlled trials exist that compare teprotumumab with IVMP, the current standard of care outside the United States. An earlier meta-analysis and matching-adjusted indirect comparison (MAIC) comparing IVMP and placebo, and teprotumumab and IVMP among patients with moderate to severe TED found teprotumumab was associated with greater improvements in proptosis and diplopia compared with IVMP, while IVMP was associated with small, typically not clinically relevant changes in proptosis and diplopia vs placebo [21]. Still, no studies directly evaluate the differential impacts of these treatments on GO-QoL scores in patients with active moderate to severe TED. To address this gap, this study used a systematic review, meta-analysis, and MAIC to compare changes in GO-QoL scores between teprotumumab and IVMP, and between IVMP and placebo.

## Materials and Methods

We conducted a systematic literature review (SLR), followed by a meta-analysis and MAIC of changes in GO-QoL in patients with TED who received teprotumumab, placebo, or IVMP treatment.

### Data Sources and Searches

#### Individual patient data

The study used deidentified individual patient data (IPD) from phase 2 (NCT01868997) and phase 3 (NCT03298867) RCTs for teprotumumab and placebo [15, 16]. The phase 2 trial included 88 patients (teprotumumab: 43; placebo: 45). The phase 3 trial included 83 patients (teprotumumab: 41; placebo: 42). Since the inclusion and exclusion criteria were consistent across the trials, the data were amalgamated to establish treatment groups comprising 84 and 87 randomized patients for teprotumumab and placebo, respectively. For IVMP studies, IPD was not available, and only data included in the published reports could be included.

### Systematic Review Search Strategy and Study Selection

A systematic review identified literature that evaluated IVMP for patients with moderate to severe active TED. PubMed and Embase were searched from database inception to April 1, 2023, using relevant keywords and controlled vocabulary, such as “methylprednisolone” OR “intravenous steroid” AND “thyroid eye disease” OR “Graves’ ophthalmopathy” OR “endocrine ophthalmopathy” OR “Graves’ orbitopathy.” Additionally, manual searches were conducted to identify any published articles not captured through electronic searches. Only human-based and English-language studies were included.

Study screening was guided by a predefined PICOS framework (population, intervention, comparator, outcomes, and study design) (Supplementary Table S1) [22]. Eligible studies must have included patients with moderate to severe active TED who underwent treatment with IVMP and reported data at baseline and at the end of the treatment period (12 weeks for IVMP and 24 weeks for teprotumumab and placebo) on the GO-QoL scale (overall and/or the 2 subscales of appearance and visual function). Titles and abstracts of the records/studies were assessed to determine eligibility.

Patients must have been treated with IVMP at a moderate cumulative dose of 4.5 g administered over a 12-week period, which is the frequently recommended treatment for moderate to severe active TED (without diplopia and/or severe proptosis) per available guidelines [2, 13]. Studies not meeting these criteria were excluded (Supplementary Table S2) [22]. Supplementary Tables S3, 4 and 5 describe the distribution of baseline diplopia, proptosis, and disease duration, respectively, extracted from the included studies [22].

### Data Extraction and Quality Assessment

A database search was conducted by N.B. Title, abstract, and full-text screening was conducted by 2 independent reviewers (M.S., P.S.). Data extraction was completed by one reviewer (M.S.) and verified by another (P.S.) using a standardized data extraction template. Extracted data elements included study characteristics (eg, inclusion and exclusion criteria), baseline characteristics, and GO-QoL data.

#### Outcome measures

The internationally validated and disease-specific GO-QoL includes an overall scale (16 questions) as well as 2 distinct subscales: appearance (8 questions) and visual function (8 questions) [9]. Data were gathered at baseline and postintervention at weeks 6, 12, and 24. Each question is assigned a score of 1, 2, or 3, indicating serious, mild, or absent limitation, respectively. Individual scores are then summed to derive a raw score. Subscale scores are transformed using the formula (raw score − 8) ÷ 16 × 100, while the overall score is transformed as (raw score − 16) ÷ 32 × 100 [9]. Scales range from 0 (complete limitation) to 100 (no limitation). An increase in the score over time compared to baseline indicates improvement, while a decrease suggests deterioration. A change of at least 6 points is deemed a clinically meaningful difference [9]. Raw data were transformed by the researchers using the aforementioned formula in cases where the eligible studies reported raw data only.

### Statistical Analysis

#### Meta-analyses

Random effects meta-analyses were employed to pool estimates of each GO-QoL outcome for the IVMP studies. Prior to conducting the meta-analyses, a qualitative evaluation of heterogeneity across studies was performed, considering factors such as study design, inclusion and exclusion criteria, baseline characteristics, and definitions of outcomes. Analyses were performed using the R software package [23].

#### Comparative analyses

Lacking direct evidence from head-to-head RCTs comparing teprotumumab with IVMP, comparative estimates were derived using MAIC [24].

Direct comparisons of change from baseline in GO-QoL between teprotumumab and placebo were derived from the pooled IPD for completeness. Four sets of comparisons were estimated:

1. Change in GO-QoL from baseline to week 24 in patients receiving teprotumumab vs change in GO-QoL from baseline to week 12 in patients receiving IVMP to be compared indirectly using MAIC analysis;
2. Change in GO-QoL from baseline to week 12 in patients receiving IVMP vs change in GO-QoL from baseline to week 24 in patients receiving placebo to be compared indirectly using MAIC analysis;
3. Change in GO-QoL from baseline to week 24 in patients receiving teprotumumab vs change in GO-QoL from baseline to week 24 in patients receiving placebo to be compared directly using IPD (pooled data from phase 2 and 3 trials); and
4. Change in GO-QoL from baseline to week 12 in patients receiving IVMP vs change in GO-QoL from baseline to week 12 in patients receiving placebo to be compared indirectly using MAIC analysis.

Comparisons between teprotumumab vs IVMP and between IVMP vs placebo were derived from IPD on change from baseline in GO-QoL for teprotumumab and placebo and the pooled change from baseline from the IVMP studies. Outcomes were assessed at week 24 for teprotumumab and at week 12 for IVMP in order to align with the approved or recommended treatment durations of these therapies (entire course) and the study periods in the reports, which is 12 weeks for the commonly recommended European Group on Graves’ Orbitopathy dose of IVMP (0.5 g/week for 6 weeks followed by 0.25 g/week for 6 weeks; cumulative dose 4.5 g over 12 weeks) and 24 weeks for teprotumumab [2, 20].

MAIC is a likelihood-based reweighting technique in which inverse probability of treatment weights are applied to the IPD to match the published marginal distribution (eg, means and SDs of continuous variables or proportions for categorical variables) of clinically important prognostic factors reported in the IVMP studies. The resulting weights were used to estimate the weighted mean change from baseline in GO-QoL for teprotumumab and placebo, which were then compared with pooled estimates of the mean change from baseline in GO-QoL for IVMP. Estimates were reported as mean differences and 95% confidence intervals (CIs).

#### Identification of prognostic factors

To enhance methodological rigor, prognostic factors used to compute weights were identified via predictive modeling of changes in GO-QoL, and expert opinion. First, trial data from 84 teprotumumab and 87 placebo patients were examined by 3 mixed-effect models to identify the key variables contributing to GO-QoL improvement [15, 16]. The dependent variables for the model included the 3 outcome measures described in *Outcome Measures,* while key covariates as independent variables included age, sex, study visit, treatment arm (teprotumumab or placebo), symptoms [Gorman diplopia scores (0-3), proptosis change (mm), spontaneous orbital pain, gaze-evoked orbital pain]. The models also included a subject-level random effect to the within-subject variance specification. Therefore, the intraclass correlation coefficient was added to the null mixed effect model (Supplementary Fig. S1) [22]. Variability between subjects was tested over the 24-week period.

Key prognostic factors were identified from this model. A clinical expert (G.J.K.) added additional prognostic factors based on clinical expertise and rank-ordered the resulting set of prognostic factors. The final set of factors used to derive the weights included severe diplopia (percent), proptosis (mean), clinical activity score (CAS; mean), smoking status (percent), baseline GO-QoL scores (mean), female sex (percent), and age (mean) (Supplementary Table S6) [22].

#### Exploratory analysis

While the primary analysis focused on the most frequently prescribed moderate dosage of IVMP (4.5 g as cumulative dose), a subsequent exploratory analysis included any IVMP dosage, ie, low (∼2.5 g), moderate (∼4.5-5 g), or high dose (∼7.5-8 g as cumulative dose) plus combination therapy (eg, IVMP and atorvastatin, IVMP with mycophenolate).

#### Sensitivity analysis

A key assumption required for inference is that all relevant prognostic factors were used to balance the patient populations via weighting. While every effort was made to adjust for the most important prognostic factors, it was not possible to adjust for CAS due to imbalances between the IPD and the IVMP trials without a substantial loss of effective sample size. As CAS was deemed to be prognostically important for predicting GO-QoL changes, a sensitivity analysis was conducted wherein weights were constructed to reweight the patient population such that the reweighted distribution of CAS from the IPD was within 0.10 SDs of the IVMP studies.

### Role of the Funding Source

These analyses and results were reviewed by the funder [Amgen Inc. (formerly by Horizon Therapeutics)], which was involved in reviewing, revising, and approving the submitted manuscript.

## Results

**Figure 1.**
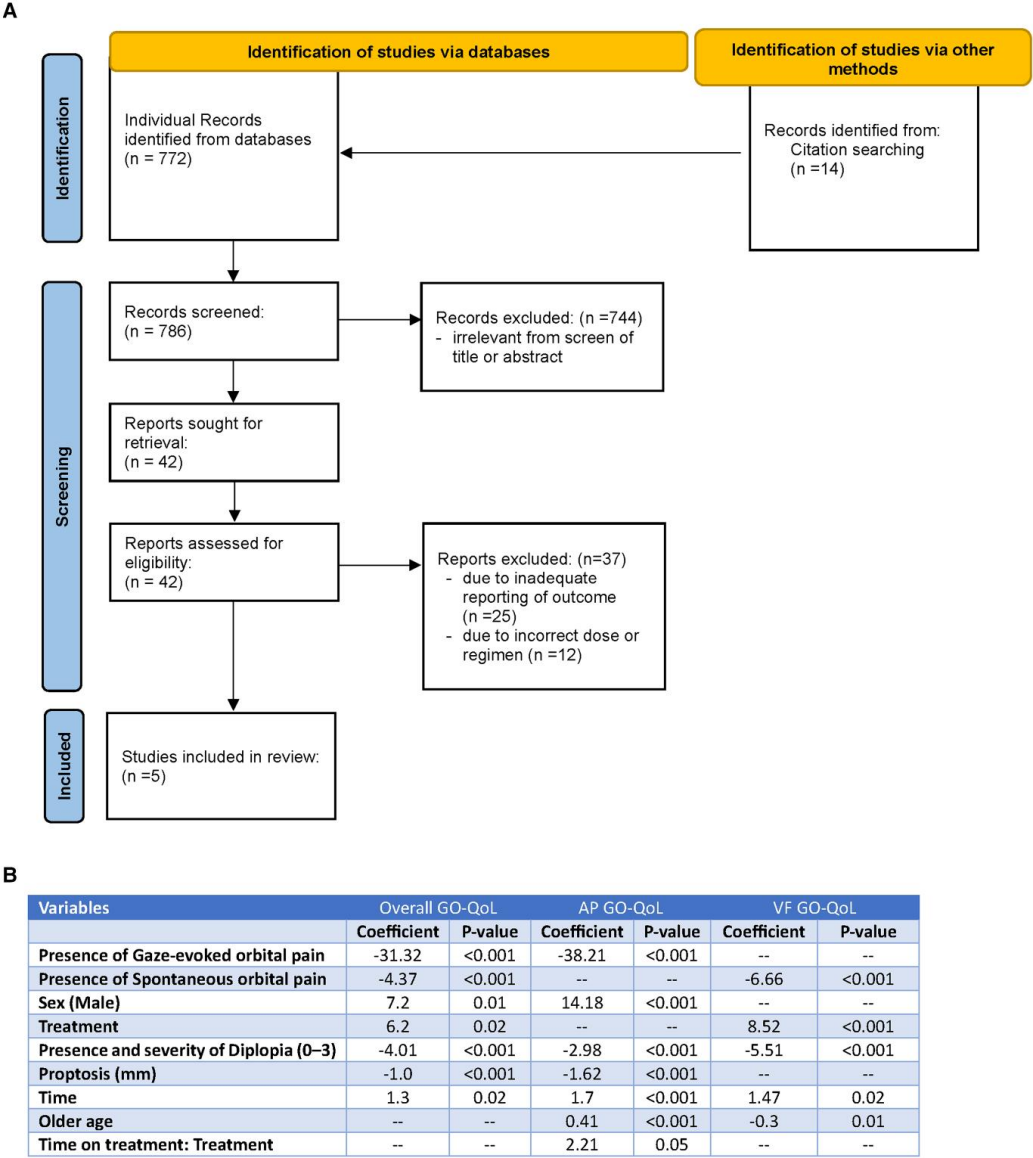
(A) Study selection. (B) Linear mixed-effects model to identify factors influencing GO-QoL score: n = 171. Using data from the study baseline, and weeks 6, 12, and 24, linear mixed-effects models were employed to measure the impact of demographics, time on treatment; proptosis (mm); diplopia (Gorman grade); presence/ absence of gaze-evoked orbital pain; and spontaneous orbital pain on total, appearance, and visual function GO-QoL scores, via hierarchical addition (Supplementary Fig. S1). Random effects accounted for within-patient response variability. “Time” refers to the time between the baseline and last visit for either teprotumumab or placebo. “Time on treatment: Treatment” refers to differences between treatment (teprotumumab and placebo) over time. Abbreviation: GO-QoL, Graves’ Orbitopathy Quality of Life Questionnaire.

Figure 1, panel A shows the selection process for the literature review for IVMP studies. The search strategy yielded a total of 772 records. Additionally, 14 records were identified through manual searches and alerts and by screening reference lists of reviewed articles, resulting in the identification of 786 total records. After removing duplicates, the titles and abstracts of records were assessed, leading to the exclusion of 744 irrelevant ones. The remaining 42 full-text records underwent detailed screening, during which 25 were excluded due to inadequate reporting of outcomes and 12 due to incorrect dose/regimen. Ultimately, 5 studies were deemed eligible for inclusion: Bartalena (2012) [12], Hoppe (2021) [25], Lanzolla (2021) [26], Kahaly (2018) [27], and Shen (2022) [28].

Details of the included studies are presented in Table 1 with further information on the characteristics of the included studies in Supplementary Table S7 [22].

**Table 1.** Characteristics of included studies focusing on IVMP as well as teprotumumab and placebo from IPD

Studies	Treatment	Analysis plan*^a^*	Study characteristics		Baseline characteristics								
			Baseline Sample size	Study design	Age	Female sex	Smokers	CAS	Baseline severe diplopia	Baseline proptosis (mm)	Baseline total GO-QoL	Baseline AP GO-QoL	Baseline VF GO-QoL
Measures	−	−	n	−	Mean (SD)	%	%	Mean (SD)	%	Mean (SD)	Mean (SD)	Mean (SD)	Mean (SD)
TEP and PBO groups from IPD													
Kahaly [20]	TEP	−	84	RCT	51.5 (11.6)	69	24	5.1 (0.9)	51.2	23.02 (3.23)	61.3 (22.3)	58.6 (27.5)	64.0 (25.8)
	PBO	−	87	RCT	51.4 (13.1)	77	30	5.3 (0.9)	35.6	23.15 (3.05)	60.1 (20.3)	55.5 (23.7)	64.8 (26.4)
IVMP group from meta-analysis													
Bartalena [12]	IVMP 2.25 g (CD)	EA	53	RCT	54 (10)	70	41	4.6 (1.1)*^b^*	47	23.3 (3.2)	60.5 (30.4)*^c^*	62 (25)	59 (30)
	IVMP 5 g (CD)	PA	54		50 (9)	57	54	4.3 (0.8)*^b^*	54	22.2 (3)	62.5 (27.3)*^c^*	70 (18)	55 (29)
	IVMP 7.5 g (CD)	EA	52		56 (11)	81	31	5.0 (1.5)*^b^*	50	22.5 (2.8)	56.5 (28.9)*^c^*	62 (23)	51 (29)
Hoppe [25]	IVMP 4.5 g (CD)	PA	100	Non-RCT	50.6 (10.7)	72	57	4.6 (2.5)*^b^*	44	21.8 (3.2)	65.1 (16.7)	63.7 (21.6)	66.5 (24.5)
Lanzolla [26]	IVMP 4.5 g (CD)	PA	39	RCT	52.4 (11.2)	77	36	4.1 (1.1)	48	23.0 (2.9)	45.9 (16.6)*^d^*	46.9 (25)*^d^*	55.6 (22.5)*^d^*
	IVMP 4.5 g (CD) and AVS 20 mg/day	EA	41		55.3 (9.2)	78	22	4.3 (1.0)	61	22.6 (2.9)	50 (17.2)*^d^*	48.1 (22.5)*^d^*	55.6 (16.9)*^d^*
Kahaly [27]	IVMP 4.5 g (CD)	PA	81	RCT	50.6 (10)	79	51	3.7 (1.3)*^b^*	41.1	21.27 (3.7)	66.1 (19.3)	63.2 (22.9)	69.8 (21.8)
	IVMP 4.5 g (CD) and MMF 720 mg/day	EA	83		52.1 (10.1)	73	53	3.7 (1.0)*^b^*	44.5	21.09 (3.5)	63.1 (16.2)	61.7 (20.7)	62.9 (21.4)
Shen [28]	IVMP 4.5 g only	PA	30	RCT	43.2 (9.7)	70	43*^e^*	4.0 (1.0)*^b^*	60	22.3 (2.3)	54.6 (29.5)*^c^*	52.5 (25.5)	56.8 (28.3)
	IVMP 3 g (reduced MP) + oral MTX	No	30		43.6 (10.7)	76.7	50*^e^*	4.0 (1.5)*^b^*	26.7	21.6 (2.1)	60.4 (26.6)*^c^*	54.2 (21.9)	66.6 (26.2)
	IVMP 4.5 g + oral MTX	No	30		45.2 (10.6)	66.7	33*^e^*	4.2 (0.8)*^b^*	43.3*^b^*	22.0 (2.7)	52.8 (27.9)*^c^*	51.9 (26.5)	53.7 (24.4)
Pooled estimates for PA*^a^*	−	−	−	−	49.99 (10.19)	71.6	50.7	4.16 (1.69)	47.1	21.99 (3.14)	61.41 (21.06)	61.42 (22.25)	62.96 (24.83)
Pooled estimates for EA*^a^*	−	−	−	−	50.97 (10.22)	72.9	45.2	4.20 (1.46)	46.6	22.05 (3.08)	59.86 (22.69)	59.80 (22.59)	60.76 (24.88)

Abbreviations: AP, appearance; AVS, atorvastatin; CAS, Clinical Activity Score; CD, cumulative dose; EA, exploratory analysis; GO-QOL, Graves’ Orbitopathy Quality of Life Questionnaire; IPD, individual patient data; IVMP, intravenous methylprednisolone; MMF, mycophenolate; MTX, methotrexate; PA, primary analysis; PBO, placebo; RCT, randomized clinical trial; TEP, teprotumumab; VF, visual function.

^a^Analysis plan: EA: exploratory analysis focusing on any IVMP dose or combination therapy; PA: primary analysis focusing on only moderate dose IVMP.

^b^Mean (SD) were imputed from reported median and Q1/Q3.

^c^Total scores were obtained from the average of the 2 standardized/transformed subscales.

^d^Reported raw scores were transformed to 100% to be consistent with the rest of the studies and the questionnaire's guideline.

^e^Smokers and passive smokers were combined.

The teprotumumab trials were placebo-controlled, double-masked, multicenter investigations conducted across Europe and the United States. Among the IVMP studies, Bartalena (2012) and Kahaly (2018) were multicentered, while others were single-center studies. Apart from Hoppe (2021), the remaining studies were randomized trials. Shen (2022) was undertaken in China and the others in the EU, Italy, and Germany.

Patient inclusion criteria across studies were largely comparable, including individuals aged ≥18 years with moderate to severe active TED. In the primary analysis, the pooled mean (SD) age of participants in the IVMP studies was 49.99 (10.19) years vs 51.50 (11.60) in the teprotumumab group and 51.40 in the placebo group from IPD (trials) (Table 1). The pooled proportion of female participants was 71.6% vs 69.0% in the teprotumumab group and 77.0% in the placebo group. The pooled mean (SD) CAS was recorded as 4.16 (1.96), compared to 5.10 (0.93) for teprotumumab and 5.30 (0.86) for placebo; the pooled mean (SD) baseline proptosis was 21.99 (3.14) mm vs 23.02 (3.23) mm for teprotumumab and 23.15 (3.05) mm for placebo. The pooled proportion of severe diplopia was 47.1% vs 51.2% for teprotumumab and 35.6% for placebo.

**Figure 2.**
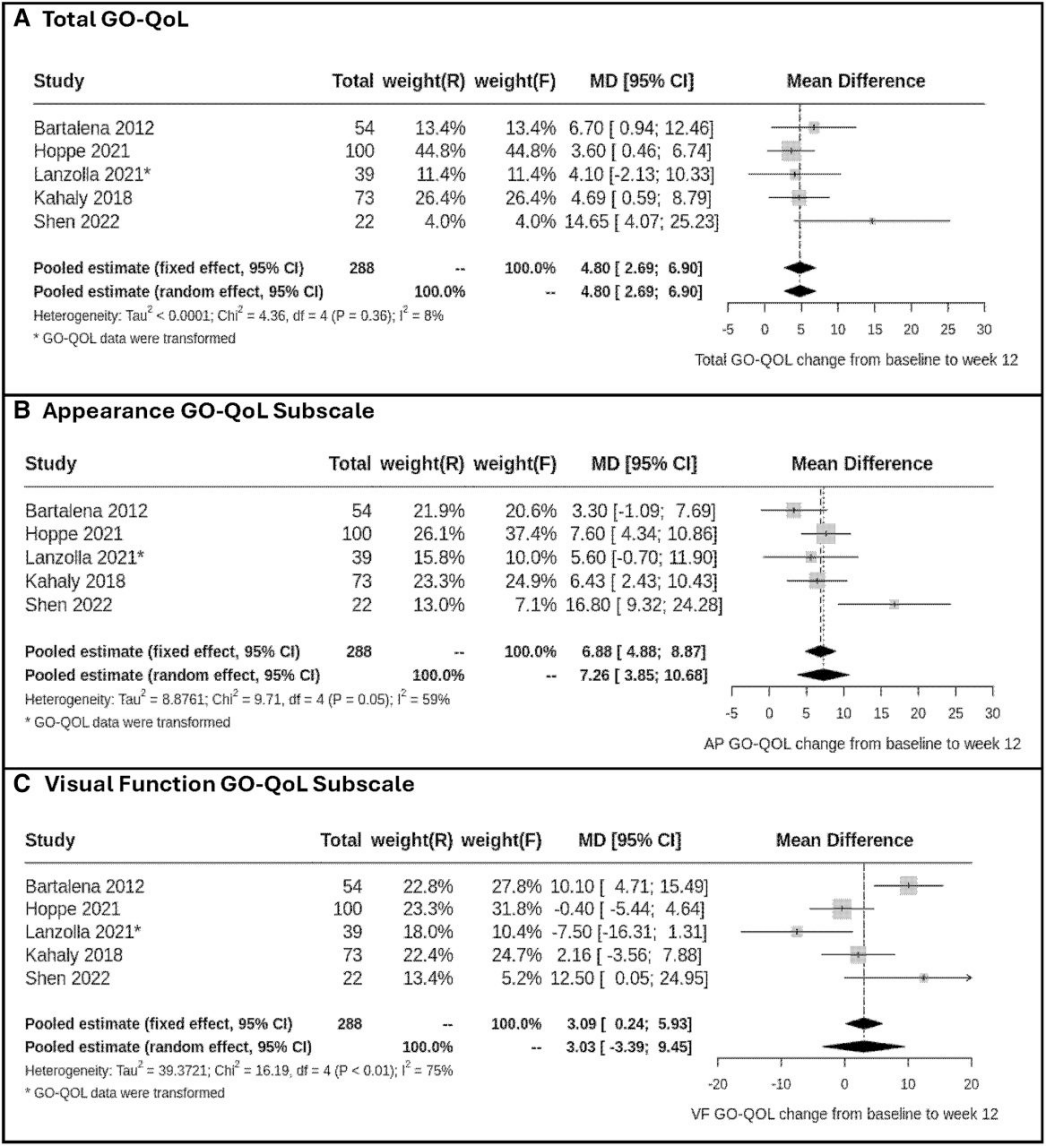
Meta-analyses to pooled estimates for moderate-dose intravenous methylprednisolone (primary analysis) for change from baseline to week 12 in GO-QoL scores. (A) Total GO-QoL; (B) Appearance GO-QoL subscale, and (C) Visual function GO-QoL subscale. Abbreviation: GO-QoL, Graves’ Orbitopathy Quality of Life Questionnaire.

Figure 2 illustrates the outcomes derived from a random-effects meta-analysis of moderate-dose IVMP, highlighting the change in score from baseline to week 12. The pooled estimate of the change score for the overall GO-QoL from baseline to week 12 showed a significant increase, with a mean difference of 4.80 (95% CI: 2.96, 6.90) (Fig. 2, panel A). Similarly, the pooled estimate of the change score in the appearance GO-QoL subscale from baseline to week 12 showed a significant increase, with a mean difference of 7.26 (95% CI: 3.85, 10.68) (Fig. 2, panel B). However, the pooled estimate of the change score in the visual function GO-QoL subscale from baseline to week 12 showed no statistical improvement, with a mean difference of 3.03 (95% CI: −3.39, 9.45) (Fig. 2, panel C).

Regarding the identification of prognostic factors, intraclass correlation coefficient results for the mixed-effects models indicated significant within-patient variability (overall: 63%; appearance: 72%; visual function: 61%). The mixed-effects model identified the following covariates as predictive of change from baseline in GO-QoL (Fig. 1, panel B): age, sex, study visit, treatment arm (teprotumumab or placebo), diplopia, proptosis, spontaneous orbital pain, and gaze-evoked orbital pain. Orbital pain (gaze and spontaneous), teprotumumab treatment, male sex, diplopia, and the presence and severity of proptosis were found to be significant independent predictors of overall GO-QoL (based on fixed β coefficients with *P* < .05). Results were similar for the appearance and visual function subscales, with some variability in the importance of predictors for visual function vs appearance (eg, spontaneous orbital pain was predictive of improved visual function but not appearance). Gaze-evoked orbital pain had the highest impact on overall and appearance GO-QoL. In addition to these covariates, a clinical expert (G.J.K.) identified 3 additional factors (smoking, baseline GO-QoL, and CAS) that were also considered to be predictive of changes in GO-QoL (Supplementary Table S6) [22]. The factors identified by the predictive model and via clinical opinion were rank-ordered according to their prognostic strength: severe (constant and/or inconstant) diplopia, proptosis, CAS, smoking, sex, age, and baseline GO-QoL (Fig. 1, panel B; Supplementary Table S6; Supplementary Fig. S1) [22]. Baseline characteristics before and after matching adjustment are provided (Supplementary Table S8) [22].

**Figure 3.**
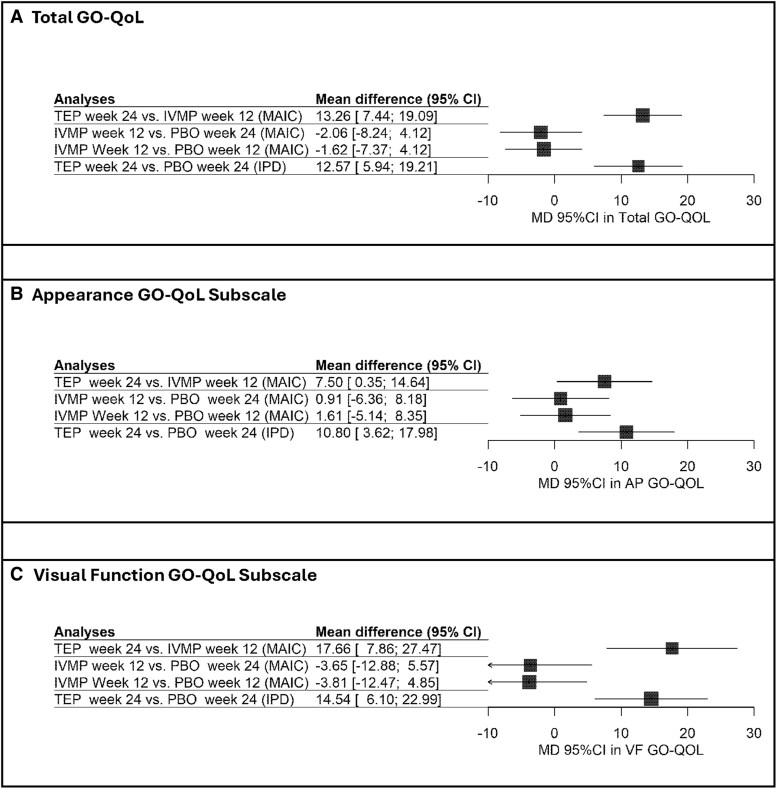
Unanchored matching-adjusted indirect comparisons and pooled results from clinical trials for change in GO-QoL scales (primary analysis). (A) Total GO-QoL, (B) Appearance GO-QoL subscale, and (C) Visual function GO-QoL subscale. Abbreviation: GO-QoL, Graves’ Orbitopathy Quality of Life Questionnaire.

MAIC for the primary analysis (moderate IVMP dose only) showed a statistically significant improvement in overall GO-QoL in patients receiving teprotumumab vs IVMP [mean difference (MD): 13.26; 95% CI: 7.44, 19.09] and placebo (MD: 12.57; 95% CI: 5.94, 19.21); however, no significant improvement was noted in overall GO-QoL when comparing IVMP against placebo (comparison of placebo data at week 24: MD: −2.06; 95% CI: −8.24, 4.12 and placebo data at week 12: MD: −1.62; 95% CI: −7.37, 4.12). Similarly, a statistically significant improvement in appearance GO-QoL subscale was identified in patients administered teprotumumab vs IVMP (MD: 7.50; 95% CI: 0.35, 14.64) and placebo (MD: 10.80; 95% CI: 3.62, 17.98), while comparing IVMP with placebo showed no statistically significant improvement (comparison of placebo data at week 24: MD: 0.91; 95% CI: −6.36, 8.18 and placebo data at week 12: MD: 1.61; 95% CI: −5.14, 8.35). Greater improvements were observed in visual function GO-QoL subscale for patients receiving teprotumumab vs IVMP (MD: 17.66; 95% CI: 7.86, 27.47) and placebo (MD: 14.54; 95% CI: 6.10, 22.99), whereas no statistically significant improvement was seen when comparing IVMP vs placebo (comparison of placebo data at week 24: MD: −3.65; 95% CI: −12.88, 5.57 and placebo data at week 12: MD: −3.81; 95% CI: −12.47, 4.85) (Fig. 3). Sensitivity analyses adjusting for CAS showed consistent results (Supplementary Table S9) [22].

Additional exploratory analyses including all IVMP doses/combination therapies produced slightly lower pooled estimates of changes from baseline in GO-QoL (Supplementary Fig. S2), resulting in smaller comparative estimates in favor of teprotumumab vs IVMP [22]. Despite these small changes, estimates still demonstrated statistically significant improvement in change from baseline in GO-QoL for teprotumumab vs IVMP, except for appearance (Supplementary Fig. S3, Supplementary Table S9) [22]. As with the primary analysis, the inclusion of CAS in the adjustment set showed consistent results for the base case and exploratory analyses (Supplementary Table S9) [22].

## Discussion

This meta-analysis has shown that compared with IVMP and placebo in the primary analysis, teprotumumab was associated with significant improvements from baseline in overall GO-QoL and the appearance and visual function subscales. In contrast, no improvement was noted in overall GO-QoL scores when comparing IVMP against placebo. These results were consistent even after considering additional IVMP doses in the exploratory analyses. Additionally, a large set of prognostic factors were identified using linear mixed modeling. The MAIC findings demonstrate that teprotumumab produces greater QoL improvement as compared with IVMP in patients with moderate to severe TED. They also suggest that IVMP provides no considerable benefit as compared with placebo.

Functional impairment and changes in appearance impact the well-being and health-related QoL of patients with TED, and this should be considered in treatment decisions. There are few studies comparing the efficacy of different treatments for TED. In the absence of direct evidence from head-to-head RCTs, MAICs are commonly employed to derive comparative efficacy estimates indirectly to support treatment decisions [24]. Using this approach, we estimated QoL improvements in patients with TED following treatment with teprotumumab compared to IVMP and for IVMP compared to placebo. Further, we examined the major factors contributing to these negative QoL changes. Furthermore, a clinical expert reviewed the results and identified 3 additional factors (smoking, baseline GO-QoL, and CAS) considered to be predictive of changes in GO-QoL. Several factors identified from linear mixed modeling were not considered for the MAIC as they were not readily available or reported on in the SLR among IVMP studies (ie, presence of gaze-evoked orbital pain).

Evidence suggests that IGF-IR overexpression by orbital fibroblasts and B and T cells is involved in TED pathogenesis [17, 18, 29]. IGF-IR forms a complex with the thyrotropin receptor, through which downstream signaling events lead to the expansion of orbital muscle and adipose tissue and consequently proptosis, diplopia, and orbital pain [19]. IGF-IR inhibition has been shown to block immune responses in TED [30]. By binding to IGF-IR, teprotumumab blocks pathogenic signaling, which is thought to lead to observed improvements in proptosis, diplopia, and inflammation [15, 16]. Consequently, improved clinical symptoms may be reflected in GO-QoL scores, which measure the impact of TED on appearance and visual function [15, 16]. In contrast, the variable effects of IVMP on proptosis and diplopia may have contributed to relatively lower GO-QoL improvements compared to what was observed with teprotumumab.

In this analysis, teprotumumab significantly improved the GO-QoL total score and the associated subscales compared to placebo. This aligns with findings from the previously published MAIC, which found teprotumumab provided greater proptosis and diplopia response vs IVMP in patients with TED [21]. Taken together, the improvements observed in QoL outcomes with teprotumumab treatment may follow its ability to improve diplopia and proptosis. Similarly, our predictive model for the identification of prognostic factors indicates that improvements in diplopia, proptosis, and pain drive improvements in QoL, with orbital pain a key determinant for QoL. While these findings support the association of QoL outcomes with diplopia and proptosis, further research is needed to identify and verify the most important clinical predictors of positive QoL outcomes.

There was variability in reported QoL outcomes across the studies investigating IVMP, which may stem from heterogeneous population characteristics such as CAS and smoking status. The MAICs conducted here adjusted for these population differences by balancing the prognostic factors and treatment-effect modifiers.

There are several limitations to this study. First, unanchored MAICs were conducted due to the lack of a common comparator arm between the included studies, as there are no major placebo-controlled IVMP studies available. The unanchored MAIC method assumes that all prognostic factors and treatment-effect modifiers are well balanced across the studies being compared. This assumption may not be met here given that characteristics considered in the MAICs are limited by not only a lack of placebo-controlled or individual patient data but also differences in the included populations (such as geography, duration of TED, severity) not accounted for and by reporting in the included studies. However, the most relevant characteristics based on data availability and literature review were used. Second, for the comparison between IVMP vs placebo and IVMP vs teprotumumab, the effective sample size was reduced when adjusting for relevant characteristics, which increases the likelihood of differences between the studies being compared, results in broad CIs, and possibly confounds the results. Only 5 IVMP studies fit the inclusion criteria and could be included. Third, the comparisons were limited to teprotumumab and IVMP, which may exclude other treatments in TED. Fourth, these analyses did not incorporate a comparison of teprotumumab with other treatments (eg, rituximab) due to a relative scarcity of high-quality RCTs, resulting in a sparse evidence network with small sample sizes. Consequently, standard network meta-analytic methods for the synthesis of evidence from all plausibly relevant comparators beyond teprotumumab and IVMP were unlikely to produce robust, high-quality evidence conducive to informing treatment decisions. Future studies should also consider the comparison of QoL improvement between teprotumumab and alternative treatments for TED.

Nevertheless, strengths of this study include it being the first to demonstrate comparative QoL evidence for teprotumumab vs IVMP. Furthermore, appropriate methodology was employed to compare data from different studies, and a TED-specific measure of QoL was used. Finally, a rigorous, multifaceted approach was used to identify and select a large set of prognostic factors that were adjusted for in the MAICs to reduce the heterogeneity among study populations and uncover those variables most correlated with severity and improvement in QoL.

In conclusion, teprotumumab was associated with statistically and clinically significant improvements in QoL, as measured by the GO-QoL, vs IVMP and placebo. Likewise, patients treated with a moderate dose of IVMP did not demonstrate statistically significant improvements in GO-QoL scores vs placebo on any of the GO-QoL scales. While this study suggests that teprotumumab results in better improvement in QoL compared with intravenous steroids, only RCTs comparing these treatments can provide a definitive determination of their differential effects on treatment outcomes. These data suggest that teprotumumab produces a greater improvement in QoL as compared with IVMP in patients with moderate to severe active TED.

## Data Availability

There is a plan to share data. This may include deidentified individual patient data for variables necessary to address the specific research question in an approved data-sharing request, as well as also related data dictionaries, study protocol, tatistical analysis plan, informed consent form, and/or the clinical study report. Data sharing requests relating to data in this manuscript will be considered after the publication date and (1) this product and indication (or other new use) have been granted marketing authorization in both the United States and Europe or (2) clinical development discontinues and the data will not be submitted to regulatory authorities. There is no end date for eligibility to submit a data sharing request for these data. Qualified researchers may submit a request containing the research objectives, the Amgen product(s) and Amgen study/studies in scope, endpoints/outcomes of interest, statistical analysis plan, data requirements, publication plan, and qualifications of the researcher(s). In general, Amgen does not grant external requests for individual patient data for the purpose of reevaluating safety and efficacy issues already addressed in the product labeling. A committee of internal advisors reviews requests. If not approved, requests may be further arbitrated by a Data Sharing Independent Review Panel. Requests that pose a potential conflict of interest or an actual or potential competitive risk may be declined at Amgen's sole discretion and without further arbitration. Upon approval, information necessary to address the research question will be provided under the terms of a data sharing agreement. This may include anonymized individual patient data and/or available supporting documents containing fragments of analysis code where provided in analysis specifications. Further details are available at https://wwwext.amgen.com/science/clinical-trials/clinical-data-transparency-practices/clinical-trial-data-sharing-request.
